# Daratumumab and Nelarabine Treatment as Salvage Therapy for T-Lymphoblastic Lymphoma: A Case Report

**DOI:** 10.3390/biomedicines12030512

**Published:** 2024-02-24

**Authors:** Gonzalo Castellanos, Laura Pardo, Alberto López, Javier Cornago, Jose Luis López, Alicia de las Heras, Francisco J. Díaz, Marta Martínez de Bourio, Eva Castillo, Pilar Llamas, Laura Solán

**Affiliations:** 1 Department of Hematology, Fundación Jiménez Díaz University Hospital, Avenida Reyes Católicos, 28040 Madrid, Spain; gonzalo.castellanos@quironsalud.es (G.C.); alberto.lgarcia@quironsalud.es (A.L.); javier.cornago@hospitalreyjuancarlos.es (J.C.); jllopez@quironsalud.es (J.L.L.); alicia.heras@quironsalud.es (A.d.l.H.); pllamas@fjd.es (P.L.); laura.solan@quironsalud.es (L.S.); 2 Department of Anatomical Pathology, Fundación Jiménez Díaz University Hospital, Avenida Reyes Católicos, 28040 Madrid, Spain; fjavier.diazp@quironsalud.es; 3 Department of Nuclear Medicine, Fundación Jiménez Díaz University Hospital, Avenida Reyes Católicos, 28040 Madrid, Spain; marta.martinezb@quironsalud.es; 4 Department of Hospital Pharmacy, Fundación Jiménez Díaz University Hospital, Avenida Reyes Católicos, 28040 Madrid, Spain; ecastillob@fjd.es

**Keywords:** lymphoblastic lymphoma, relapse, salvage therapy, nelarabine, daratumumab

## Abstract

T-cell lymphoblastic lymphoma is an uncommon lymphoid neoplasm in adults, although more frequent in children and teenagers, that often affects the mediastinum and bone marrow, requiring intensive chemotherapy protocols. Its prognosis is poor if a cure is not achieved with first-line treatments. We present a case report of a 19-year-old man diagnosed with this type of lymphoma due to significant respiratory distress and a mediastinal mass. He received treatment according to the hyper-CVAD regimen, with a complete metabolic response. However, seven months later a new mediastinal growth was observed, leading to salvage treatment with a combination of nelarabine and daratumumab. We observed not only refractoriness, but also leukemization, which prompted consideration of hematopoietic stem cell transplantation. Based on this case, we conducted a review of pharmacological treatment options for refractory or relapsed lymphoblastic lymphoma, as well as the role of radiotherapy in managing mediastinal disease. This case report highlights the limited evidence available regarding later-line treatments, with unusual reports regarding employing our combination of daratumumab and nelarabine, and emphasizes the importance of achieving cures in the first line of treatment.

## 1. Introduction

T-cell lymphoblastic lymphoma (T-LLy) is a lymphoid neoplasm that comprises 25% of childhood non-Hodgkin lymphomas (NHL) and 3% of those in adults, usually originated from early T-cell progenitors [1,2]. It is a rare disease, but its incidence seems to be increasing over time, typically consisting of a mediastinal mass and partial bone marrow involvement [3,4]. Although there are intensive chemotherapy protocols for high-risk cases aimed at a curative approach for initial management, second-line treatments are not as well-defined. Nelarabine (a deoxyguanosine analog, which is toxic to T lymphocytes leading to the inhibition of DNA synthesis) [5] and daratumumab (a human IgG1κ monoclonal antibody that binds to a CD38 epitope) [6] have been previously employed with diverse results, though stronger evidence supports their combined administration in current studies. 

The use of intensive chemotherapy followed by allogenic hematopoietic stem cell transplantation (HSCT) as a consolidation treatment has been reported in many cases of adverse prognosis and relapsed or resistant disease. Although some data suggest that this treatment could be an effective alternative that could improve relapse-free survival, and even offer better outcomes than only chemotherapy for high-risk patients, its role remains controversial [2,7].

## 2. Clinical Case

A 19-year-old male with no relevant medical history was admitted to the Emergency Room at the end of September 2022, with dysphagia and persisting dyspnea for approximately three weeks. These were attributed to a cervical mass with apparent vascular invasion and tracheo-esophageal contralateral displacement, as well as left internal jugular vein thrombosis. 

**Figure 1:**
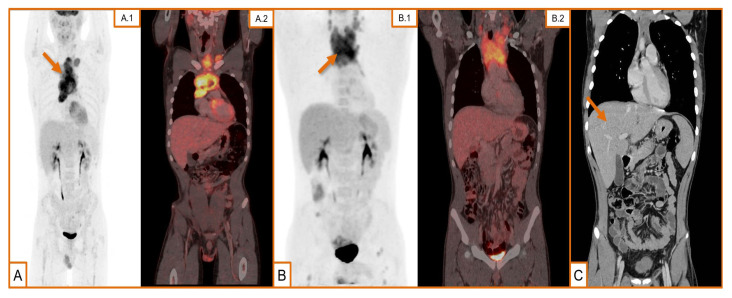
Radiological findings in our case evolution. (**A**) (**A.1**) Maximum intensity projection (MIP) image of positron emission tomography (PET) using 18F-Fluorodeoxyglucose (18F-FDG) as radiotracer. Hypercaptant mediastinal mass (arrow) with heterogeneous distribution suggestive of thymoma/lymphoproliferative syndrome at the time of diagnosis; (**A.2**) fusion images, sagittal axe. (**B**) (**B.1**) MIP image of PET using 18F-FDG as tracer. Supradiaphragmatic and cervical adenopathy clusters with a bulky mediastinal mass (arrow) and pathological FDG bone deposits in both femurs during the first relapse; (**B.2**) fusion images, sagittal axe. (**C**) Remarkable reduction in the size of the mediastinal mass, although newly observed hepatomegaly (arrow) after salvage therapy.

A PET-CT scan [Figure 1A] subsequently revealed that the previously known mass, likely of thymic origin, displayed abnormal FDG uptake and associated lymphadenopathy. After undergoing a thoracoscopic biopsy in November 2022, the diagnosis of T-LLy was confirmed. In this first diagnostic sample, cytogenetics did not show any remarkable chromosomic alteration, and next-generation-sequencing (NGS) was not performed due to the lack of enough sample. We also performed a bone marrow biopsy that confirmed lack of disease infiltration and normal hematopoiesis.

One week later, the patient returned to the Emergency Room due to pronounced respiratory distress leading to respiratory failure caused by compression of the airway exerted by the thoracic mass, and necessitating a 72 hours admission in the Intensive Care Unit. Given the previously mentioned lymphoma diagnosis, the patient was started on a prephase regimen involving cyclophosphamide and prednisone. Following a lumbar puncture that ruled out central nervous system (CNS) disease infiltration, intrathecal chemotherapy with methotrexate, cytarabine, and dexamethasone was administered, thereby marking the start of the patient’s therapeutic protocol with the high-intensity hyper-CVAD scheme. First cycles were administered without significant incidents, during which multiple lumbar punctures were performed, with none showing lymphomatous infiltration of the CNS.

By the end of February 2023, a follow-up CT scan was performed after the first part of the third cycle of hyper-CVAD, which confirmed the achievement of a complete response. However, due to persistent symptoms related to a previous flu-like episode, the patient was readmitted two weeks later, and following a diagnosis of respiratory sepsis, the decision made was to withhold the final phase of the third cycle of hyper-CVAD, and a PET-CT scan was requested for end-of-treatment evaluation.

**Figure 2:**
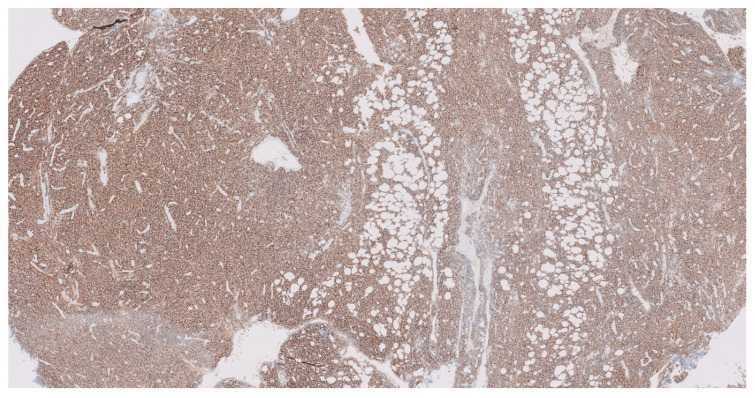
Relapse mediastinal mass biopsy, 10× photography. Immunohistochemical analysis revealed a distinctly positive expression for CD38 (transmembrane glycoprotein and a marker of lymphocyte differentiation and activation) in tumoral cells’ surfaces in the excisional biopsy of the mediastinal mass, conducted subsequent to radiological confirmation of relapse.

This PET-CT scan [Figure 1B] revealed the growth of the previous mediastinal mass and multiple lymph node conglomerates, so the patient was readmitted under the suspicion of relapse. An excisional biopsy was performed [Figure 2], with pathology results that confirmed our hypothesis. Overexpression of *p53* was identified in immunohistochemistry, cytogenetics were again negative, and NGS showed a pathogenic variant of GATA3^R363Pfs*39^ and another of uncertain significance in *BCOR^A220V^*. 

Salvage treatment was then considered, using a proposed combination of nelarabine and daratumumab. Radiation therapy was added as well, as a more direct approach to the mediastinal mass. While waiting for definitive results, further prephase treatment with corticosteroids was initiated, resulting in an excellent response and clinical improvement.

The administered rescue regimen chosen as a second line of treatment eventually consisted of radiotherapy, nelarabine, and daratumumab. Sixteen sessions of radiotherapy were administered and daratumumab was approved to be administered on a weekly basis until a potential HSCT could be performed. HLA-patient analysis was undertaken immediately following the diagnosis, considering HSCT as a potential consolidation therapy. The patient’s mother was chosen as a donor, given the absence of other viable alternatives after a preliminary search and study of direct relatives. A follow-up CT scan [Figure 1C] after the initial rescue cycle indicated a notable reduction in the cervical mass, albeit hepatomegaly, with signs of portal hypertension described for the first time. 

Five days after CT scan results were delivered, the patient consulted at the Emergency Department with high fever and clinical deterioration. A blood smear analysis revealed leukemization of the underlying disease, with a blast count of 74%, and worsening hepatic function. A hepatic transjugular biopsy confirmed liver infiltration. The patient also had mandibular paresthesia, which suggested nervous system involvement due to CNS infiltration. However, once again, the cerebrospinal fluid study was negative. 

Therefore, corticosteroid treatment was initiated once again, and a search for clinical trials in other centers was conducted, which yielded no alternatives to a sequential HSCT. 

The patient was informed about the poor prognosis of this option, and he eventually opted for symptomatic management and palliative care through a home care unit. Ultimately, approximately two weeks later, the patient passed away.

## 3. Discussion

T-lymphoblastic lymphoma is a notably aggressive clinical entity, a fact that can be inferred from our case’s chronology, wherein a mere lapse of eight months passed from the diagnosis to decease. Historically, treatment schemes have consisted of conventional lymphoma therapeutic regimens, resulting in extremely poor outcomes. Most of these cases exhibited primary refractoriness or close relapses [2,4].

More aggressive NHL protocols manifested improved response rates, but failed to improve survival. Investigations demonstrated that although both intensive and prolonged chemotherapy, as well as CNS prophylaxis, were necessary to improve treatment outcomes, lymphoma standard schemes were not enough to enhance them [4]. In addition, it was noted that obtaining a complete response, even if delayed, was correlated with higher survival odds [8]. Based on these facts, induction regimens similar to those used in acute lymphoblastic leukemia (ALL) were employed, showing promising results [2].

However, it is important to remark that although both entities share some characteristics, they present differences in terms of immunophenotypes and molecular profiles, which suggests the potential benefits of more personalized approaches [1,9].

**Figure 3:**
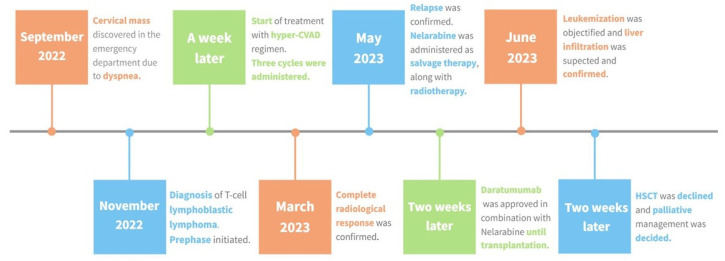
Treatment sequence. Timeline illustrating the progression of disease from diagnosis to the patient’s end. As described above, the initial approach involved the administration of the hyper-CVAD regimen as our first-line treatment, which resulted in a complete radiological response. Upon confirming the relapse, nelarabine was administered, followed by the addition of daratumumab and mediastinal radiotherapy. Following the second progression, a prephase was initiated, and HSCT was considered; ultimately the patient opted for palliative care.

The prognosis following relapses of T-LLy is adverse, with salvage rates that rarely exceed 15%. Consequently, achieving a cure in the first line of treatment is extremely important [1]. In the present case, the hyper-CVAD regimen, proposed by MD Anderson and frequently used to treat this condition [2,10], was chosen [Figure 3]. 

The integration of bortezomib within first-line therapy has been studied in clinical trials (most recently in AALL1231). It significantly improved event-free survival and overall survival (OS) of patients with T-LLy, with significant reductions in relapse and disease progression [11]. Bortezomib is generally well-tolerated and effective, and some groups recommend its use in both induction and maintenance protocols [1]. Data regarding the management of patients with relapsed or refractory disease remain limited. The use of bortezomib is considered, if not used, in the first line of treatment [1]. 

Furthermore, nelarabine is the only drug specifically approved for the treatment of patients with relapsed or refractory T-LLy [12]. It has been used in combination and as a single agent, with clear differences in therapeutic outcomes; the observed OS supports the use of combined treatment in some series [13], although its benefit appears to be limited to its use in salvage therapy, with results similar to those of conventional chemotherapy in frontline schemes reported in different studies [10]. Monotherapy responses to nelarabine range between 0 and 44% in relapsed/refractory T-LLy cases [2]. 

Nevertheless, complete response rates are not higher when compared with monotherapy, which raises certain doubts about the best strategy for nelarabine use. Nelarabine’s primary toxicity is neurological, with low-grade neuropathy, as well as hematological manifestations such as neutropenia and thrombocytopenia [5,12]. Using cyclophosphamide or etoposide in addition to nelarabine increases its hematological toxicity and, as has been stated, remains controversial [1,5].

Daratumumab is an option to be considered due to high CD38 expression on the cell membranes of T-lymphoblasts, which seems to persist even post-treatment without leading to downregulation processes and avoiding a loss of response to this drug [14], although it should be noted that this persistence seems to be lower than the observed in multiple myeloma (MM) cases [15]. Studies conducted regarding MM suggest that daratumumab’s effectiveness could correlate with the expression of CD38 found in the tumor [16]. The regimen involving daratumumab and nelarabine seems to offer some advantages as salvage therapy according to some studies, but experience in this area is clearly limited by this regimen’s use in only a few cases or to treat T-ALL [16,17]. Its broad spectrum of activity offers a treatment opportunity in other lymphoid processes beyond MM [6], showing tumor burden reduction in animal models using daratumumab. 

There are other alternatives that can be used prior to HSCT to treat relapsed or refractory disease. Venetoclax, either alone or in combination with CDK4/6 inhibitors or navitoclax, has achieved complete responses in combination with chemotherapy. Alemtuzumab (a CD52 targeting antibody) has been tested with limited response and significant toxicity due to its substantial lymphodepleting activity. Brentuximab vedotin may be considered for use in patients with significant tumor expression of CD30 [1,4]. 

Finally, it is important to emphasize that in cases in which there is any molecular alteration that can be targeted with an available drug, its use is recommended [1]. Clinical trials are considered a first approach in the treatment of these patients, but its access is limited. In our clinical case, this option was considered at the first relapse, but after a preliminary search, we found clinical trial slots only for T-ALL treatment, excluding lymphoma.

Recommendations for T-LLy second-line treatment (of refractory or relapsed cases) have been summarized in Table 1 to facilitate the reader’s access to them.

After confirming the expression of CD38 in our patient, we decided to start a combination treatment regimen of nelarabine and daratumumab. 

The presence of a residual mediastinal tumor is one of the primary reasons for partial response in patients with T-LLy. Therefore, CT-guided radiotherapy (30–36 Gy in 15–20 fractions) is an effective local treatment option that could reduce relapses in selected patients with a significant mediastinal tumor burden [2]. Its primary role appears to be part of the consolidation treatment following chemotherapy induction, and its routine use for managing residual mediastinal disease is not supported. However, the development of cardiac disease or secondary pneumonitis as complications should be monitored, and controversial results compel individualized use [4]. 

Nevertheless, further studies are needed to clarify these aspects and to identify the best alternative relapse treatment for patients who are refractory to conventional chemotherapy [1]. For patients with refractory or relapsed disease, as well as those with high-risk features, achieving a complete response followed by consolidation with a HSCT from the best available donor stands as the best approach [2,4]. However, it is crucial for the patient to maintain a functional state to tolerate the procedure. 

Relapses following HSCTs show poor prognosis, being favored by the tumor status (whether in complete response or not) prior to transplantation [2,4]. There is no actual evidence supporting the performance of HSCT as a first-line treatment or after achieving the first complete response, although there are a few studies that have assessed the use of allogeneic or autologous transplantation as consolidation treatment in such cases. The superiority of allogeneic transplantation seems to be supported by the graft-versus-host effect characteristic of this modality, which may facilitate the control of residual disease in a manner analogous to acute leukemias [7]. 

Although these series suggest favorable long-term outcomes with bone marrow transplantation in cases of chemo-sensitive disease (a category we probably cannot include our case in, given the short duration of the response achieved after chemotherapy discontinuation) and after achieving a complete response, making it an effective consolidation strategy [7], patient selection in these studies and the presence of sometimes contradictory criteria for eligibility do not allow us to currently consider it as a routine first-line treatment. As we have previously described, HSCT is therefore relegated to the treatment of refractory or relapsed cases until we have more experience with or evidence about its early use [18].

Although it was an initial consideration, and he had a haploidentical donor, our patient ultimately declined the HSCT option due to concerns surrounding uncertain results and the extensive disease involvement; also a complete response had not been achieved, which could have compromised our results.

## 4. Conclusions

T-LLy is an aggressively evolving entity, often characterized by fast progression, as observed in our case. Therefore, achieving a complete response in the first line of treatment and early identification of chemotherapy-resistant cases are significant, in order to choose the most suitable treatment for each patient. We aimed for an early and marked response to the combination of nelarabine and daratumumab, but also observed a rapid loss of response to it, which did not allow us to perform other therapies, such as HSCT. 

It is important to note that in our case, despite obtaining a complete radiological response with the first line of chemotherapy treatment, this treatment could not be completed due to infectious complications, which led to a rapid relapse. Despite not finding typical related genetic alterations, we identified overexpression of *p53*, which would, per se, condition a more aggressive phenotype of this pathology. Furthermore, the poorly maintained response to all treatments received likely categorizes ours as a resistant case, with no options available other than attempting a sequential HSCT. 

However, despite presenting a second relapse, the rapid response to our salvage therapy and the conducted review suggests the combined use of nelarabine and daratumumab as a viable rescue option in patients with limited response to conventional treatment. The use of this scheme involving proteasome inhibitors and anti-CD38 appears promising, but data are currently limited, and more clinical research is required. Radiotherapy can be considered as an adjuvant treatment and as a direct approach to mediastinal disease, but its use must be individualized. 

## Figures and Tables

**Table 1:** Summary of therapeutic options that can be considered for T-LLy second-line treatment of refractory or relapsed cases, although individualization must be taken into consideration based on the patient’s characteristics.

Recommendations for T-LLy in refractory or relapsed cases
1. Clinical trials are preferred when available [1].
2. Nelarabine is recommended as a second approach, alone or in combination with etoposide and cyclophosphamide [1,4].
3. Besides nelarabine, whenever possible, combination with a second drug is suggested: - If it has not been received as a first-line treatment, Bortezomib can be added [1]. - If CD38 expression persists on the surface of T-cell Lymphoblasts, Daratumumab can be added [1]. - If CD30 expression appears, Brentuximab vedotin can be added [4]. - Venetoclax and CDK4/6 inhibitors could be considered if previous alternatives have been discharged [1].
4. If molecular profiling is available and there are any targeted agents appropriate, they are recommended [1].
**These alternatives should be followed by HSCT as consolidation in second response if possible** [18].

## Data Availability

No new data were created or analyzed during this study. Data sharing is not applicable to this article.
